# *Pseudomonas aeruginosa*-mannose-sensitive hemagglutinin inhibits pancreatic cancer cell proliferation and induces apoptosis via the EGFR pathway and caspase signaling

**DOI:** 10.18632/oncotarget.12844

**Published:** 2016-10-24

**Authors:** Xi Cheng, Bingrui Wang, Zhijian Jin, Ding Ma, Weiping Yang, Ren Zhao, Xiaoqian Jing, Baiyong Shen, Chenghong Peng, Weihua Qiu

**Affiliations:** ^1^ Department of General Surgery, Ruijin Hospital, Shanghai Jiao Tong University School of Medicine, Shanghai, 200025, China; ^2^ Shanghai Institute of Digestive Surgery, Ruijin Hospital, Shanghai Jiao Tong University School of Medicine, Shanghai, 200025, China

**Keywords:** pancreatic cancer, PA-MSHA, cell cycle arrest, apoptosis, EGFR

## Abstract

*Pseudomonas aeruginosa*-mannose-sensitive hemagglutinin (PA-MSHA) has demonstrated efficacy against several solid tumors. In this study, we found that PA-MSHA inhibited the proliferation of PANC-1 and SW1990 pancreatic cancer cells, but had no obvious effects on HPDE6-C7 normal human pancreatic duct epithelial cells. Electron microscopy revealed the presence of apoptotic bodies and intracellular vacuole formation in PA-MSHA-treated pancreatic cancer cells. Flow cytometric analysis indicated the rate of apoptosis correlated with the PA-MSHA concentration. We observed a decrease in cell fractions in G_0_/G_1_ and G_2_/M phases, and an increase in the fraction in S phase (p < 0.01). PA-MSHA thus caused cell cycle arrest. Increasing concentrations of PA-MSHA did not alter total levels of EGFR, AKT or ERK, but levels of the corresponding phosphoproteins decreased. PA-MSHA also reduced tumor volume in a xenograft mouse model of pancreatic cancer (p < 0.01). Furthermore, caspase-3 levels decreased while the levels of cleaved caspase-3 increased (p < 0.01). These data suggest that by blocking cell cycle progression, PA-MSHA induces apoptosis and inhibits tumor growth. PA-MSHA-mediated inhibition of EGFR signaling and activation of the caspase pathway may play an important role in the induction of apoptosis in pancreatic cancer cells.

## INTRODUCTION

Pancreatic cancer is a malignant neoplasm of the digestive system. The incidence has increased and it is now the 10^th^ most prevalent malignant tumor worldwide [1–3]. The median survival time of pancreatic cancer patients is 3–6 months and the 5-year survival rate is less than 5%. Because pancreatic cancer does not exhibit obvious clinical symptoms in the early stages, it is frequently diagnosed at a late stage (85% of patients) when surgical treatment is no longer effective [4]. Therefore, adjuvant therapy (particularly radiotherapy and chemotherapy) is critical for management.

Gemzar (gemcitabine) single-agent chemotherapy is the standard treatment for metastatic or locally advanced pancreatic cancer, but it has a modest survival benefit [5,6]. FOLFIRINOX, a combination of chemotherapeutic agents (folinic acid, fluorouracil, irinotecan, and oxaliplatin), was recently shown to nearly double the median survival of pancreatic ductal adenocarcinoma (PDAC) patients compared to gemcitabine (11.1 vs. 6.8 months). However, patients must be carefully selected and managed because of toxicity [7]. Nab-paclitaxel (NPT) is a water-soluble, cremophor-free, albumin-bound formulation of paclitaxel. It was initially developed to circumvent the toxicity associated with cremophor, which is required to solubilize paclitaxel. NPT in combination with gemcitabine resulted in a median survival time of 8.5 months compared to 6.7 months after gemcitabine treatment alone [8–9]. Given the moderate improvements in PDAC patient prognosis, there is an urgent requirement for novel therapeutic strategies to improve overall survival.

Previous studies have demonstrated that *Pseudomonas aeruginosa*-mannose-sensitive hemagglutinin (PA-MSHA) in combination chemotherapy functions through mannose-modified proteins to inhibit cancer cell proliferation, induce apoptosis, and promote an anti-tumor immune response. PA-MSHA also has displayed anti-tumor activity in breast, liver, lung, gastric, and bladder cancer [10–12]. EGFR overexpression and/or enhanced activity has been reported in many solid tumors including pancreatic cancer [13]. Activation of EGFR results in phosphorylation and activation of downstream signaling components and leads to cell proliferation [14]. In this study, we investigated the mechanisms underlying PA-MSHA function. We assessed whether PA-MSHA induced apoptosis in pancreatic cancer cells, and evaluated the association between PA-MSHA-mediated anti-tumor effects and EGFR signaling. Additionally, we evaluated the therapeutic potential of PA-MSHA.

## RESULTS

### PA-MSHA inhibits pancreatic cancer cell proliferation

**Figure 1:**
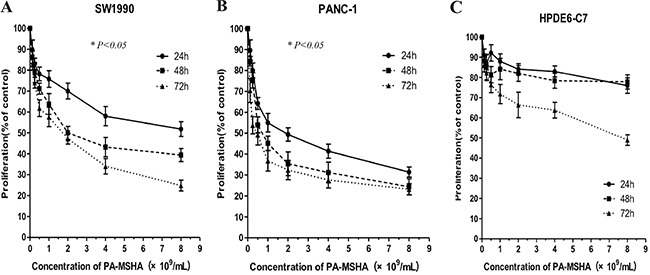
The effects of PA-MSHA on cell proliferation Values represent the percentages of untreated control cells. The data are averages of triplicate results from representative experiments. P < 0.05 for (**A**) SW1990 or (**B**) PANC-1 cells treated with PA-MSHA vs. controls. P > 0.05 for (**C**) HPDE6-C7 cells treated with PA-MSHA vs. controls.

We analyzed the effects of PA-MSHA on the proliferation of pancreatic cancer and normal ductal epithelial cell lines using CCK-8 assays. PA-MSHA at concentrations of 0.125, 0.25, 0.5, 1.0, 2.0, 4.0, 8.0 × 10^9^/mL inhibited the growth of PANC-1 and SW1990 pancreatic cancer cells in a time- and dose-dependent manner. In addition, treatment of HPDE6-C7 normal pancreatic ductal epithelial cells with PA-MSHA for 24 h and 48 h had little/no effect on proliferation (Figure 1). The IC_50_ values for the two pancreatic cancer cell lines were significantly higher than those of the normal pancreatic ductal epithelial cells (Table 1) (p < 0.05).

**Table 1:** IC_50_ values of PA-MSHA in various pancreatic cell lines

Cell lines	IC_50_(×10^9^/ml)		
	24h	48h	72h
PANC-1	1.91±0.25	0.95±0.13	0.46±0.08
SW1990	9.02±2.02	2.69±0.35	1.43±0.13
HPDE6-C7	102.60±10.87	42.24±7.63	11.88±4.32

PA-MSHA, Pseudomonas aeruginosa-mannose-sensitive hemagglutinin;

IC50, 50% inhibitory concentration.

### PA-MSHA induces changes in cellular ultrastructure

**Figure 2:**
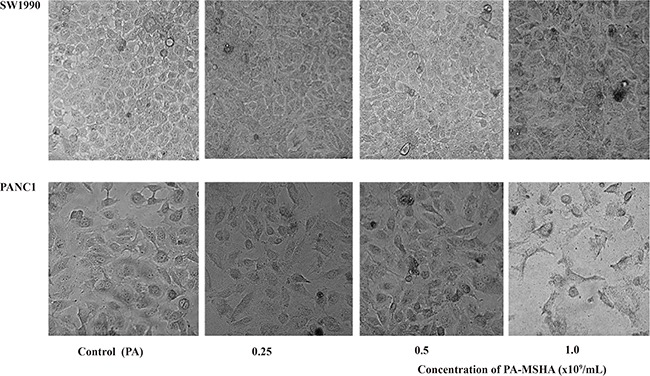
Morphological changes induced by PA-MSHA in pancreatic cancer cell lines Phase-contrast microscopy images showing the morphology of each group of cells after treatment with varying concentrations of PA or PA-MSHA (0.25, 0.5, and 1.0 × 10^9^/mL).

**Figure 3:**
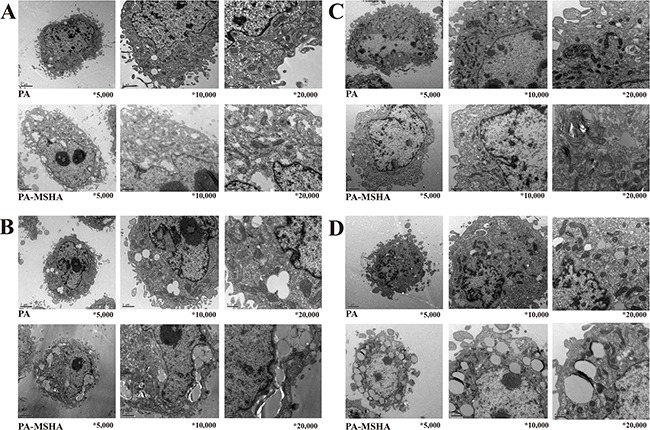
PA-MSHA-induced changes in cellular ultrastructure Changes in PANC-1 cell ultrastructure after treatment with PA-MSHA (10 × 10^8^/mL) for 24 h **A.** and 48 h **B.** visualized by electron microscopy (×5,000), (x10,000), and (x20,000). Changes in SW1990 cell ultrastructure after treatment with PA-MSHA (10 × 10^8^/mL) for 24 h **C.** and 48 h **D**. visualized by electron microscopy (×5,000), (x10,000), and (x20,000).

We next examined changes in cellular ultrastructure in response to treatment of pancreatic cancer cells with PA-MSHA by transmission electron microscopy. Treatment of PANC-1 and SW1990 cells with 0.5 × 10^9^/mL PA did not alter the cellular morphology. However, treatment with 0.5 × 10^9^/mL PA-MSHA for 24 hours resulted in cytoplasmic condensation, shrinkage of the cell body, nuclear fragmentation, mitochondrial enlargement, and the detachment and fusion of the endoplasmic reticulum with the plasma membrane to form multiple vacuoles. The presence of budding apoptotic bodies was indicative of the initiation of cellular breakdown in response to PA-MSHA treatment. Vacuole size and number increased after 48 hours of PA-MSHA treatment. At this time-point, most of the cells had died and the cytoplasmic structures had collapsed (Figure 2 and Figure 3).

### PA-MSHA induces apoptosis in pancreatic cancer cell lines

**Figure 4:**
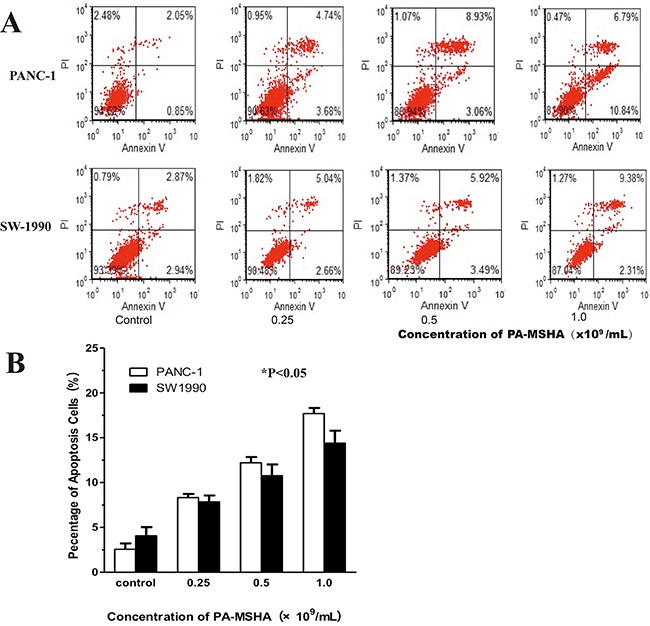
Flow cytometric analysis of apoptosis in response to PA-MSHA treatment of pancreatic cancer cells **A.** The ratio of apoptotic cells (i.e. the annexin V-positive/PI-negative fraction) was measured in PANC1 and SW-1990 cells after treatment with the indicated concentrations of PA-MSHA in serum-free media for 24 h. The results are representative of three independent experiments. **B**. The percentage of apoptotic cells showing the annexin V-positive/PI-negative fraction. The columns represent the mean ± SD of the three independent experiments. *p < 0.05 for PA-MSHA vs. controls.

The percentage of apoptotic PANC-1 and SW1990 cells was higher after PA-MSHA treatment compared to the controls (Figure 4). The rates of apoptosis were 8.17 ± 0.53% and 7.62 ± 0.89% in PANC-1 and SW1990 cells, respectively, after treatment with 0.25 × 10^9^/mL PA-MSHA. These rates were much higher than those observed in the control group (4.18 ± 1.12% and 2.49 ± 0.74% for PANC-1 and SW1990 cells, respectively). Furthermore, the rate of apoptosis increased with increasing concentrations of PA-MSHA. Thus, our data demonstrate that PA-MSHA induces apoptosis in pancreatic cancer cell lines.

### PA-MSHA blocks cell cycle progression in pancreatic cancer cell lines

**Figure 5:**
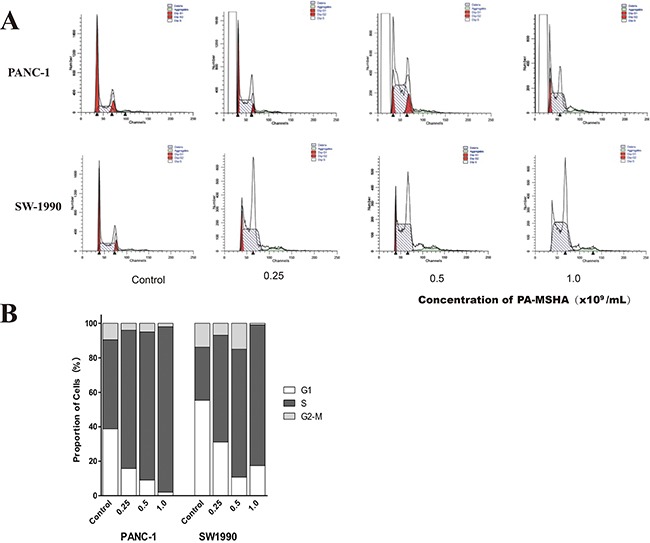
Flow cytometric analysis of cell cycle progression in human pancreatic cancer cells treated with PA-MSHA **A.** The columns represent the mean ± SD of the three independent experiments. PANC-1 and SW-1990 were treated with the indicated concentrations of PA-MSHA in serum-free medium for 24 h. The results are representative of three independent experiments. **B.** The percentages of cells in G1, S, and G2-M are shown as histograms

We next analyzed cell cycle progression in PANC-1 and SW1990 cells after treatment with PA-MSHA by flow cytometry. After 24 hours of PA-MSHA treatment, the population of pancreatic cancer cells in both the G_0_/G_1_ and G_2_/M phases was significantly lower than in the control group, while the population of cells in in S phase was significantly higher (p < 0.01) (Figure 5). These data indicated that PA-MSHA caused cell cycle arrest in S phase and induction of apoptosis.

### PA-MSHA inhibits EGFR signaling in pancreatic cancer cells

**Figure 6:**
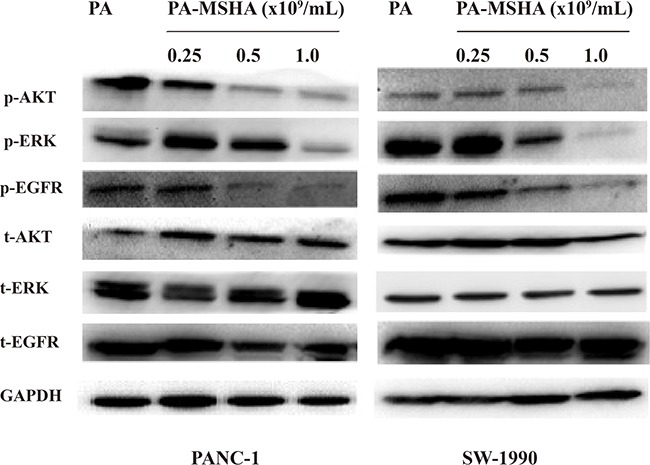
The effect of PA-MSHA on the levels of total and phosphorylated AKT, ERK, and EGFR Levels of total and phosphorylated AKT, ERK, and EGFR in PANC-1 and SW-1990 cells treated with PA-MSHA.

To evaluate the effects of PA-MSHA on EGFR signaling in pancreatic cancer cells, we examined the expression of key downstream proteins in the EGFR pathway (t-EGFR, t-AKT, t-ERK, p-EGFR, p-AKT, and p-ERK) by western blotting. Interestingly, the levels of p-EGFR, p-AKT, and p-ERK decreased with increasing concentrations of PA-MSHA, while total EGFR, AKT, and ERK protein levels did not change (Figure 6). Thus, PA-MSHA inhibited EGFR signaling in pancreatic cancer cells by blocking phosphorylation of several downstream effectors of EGFR.

### PA-MSHA and NPT have anti-tumor activity in PANC-1 tumor xenografts

**Figure 7:**
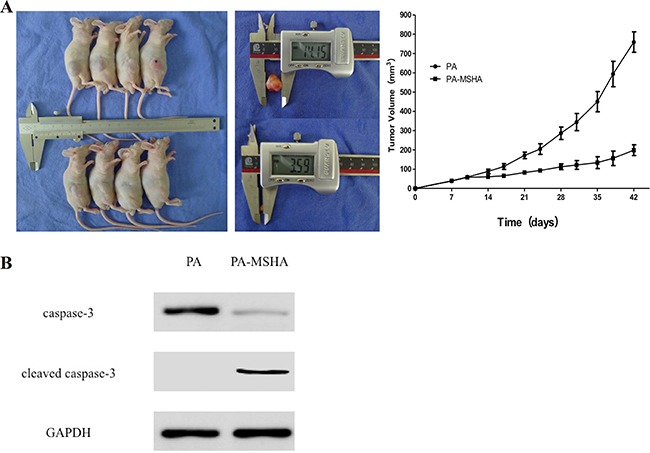
Inhibition of tumor growth in vivo by PA-MSHA **A.** Tumor volume measured at the indicated time-points. Treatment was initiated 10 days after implantation of pancreatic cancer cells into the mammary fat pads of mice. The mice were injected with either PA or PA-MSHA for 7 weeks and the tumor volumes measured. *p < 0.01. **B.** Caspase-3 and cleaved caspase-3 expression in xenograft mice treated with either PA or PA-MSHA.

To examine the anti-tumor effects of PA-MSHA *in vivo*, we subcutaneously injected nude mice with PANC-1 cells into the right hind limb. The mice were then injected intraperitoneally with NPT for 7 weeks. The mice were euthanized 42 days after transplantation and the tumor tissue collected. We found that the average tumor volume was larger in the control group than in the PA-MSHA group (714.51 ± 101.73 mm^3^ vs. 187.64 ± 28.58 mm^3^, p < 0.01). Overall, the tumors in the PA-MSHA treatment group were significantly smaller than those in the control group (Figure 7B). Finally, an additive effect was observed when the two agents were combined (Supplementary Figure 1A). Thus, PA-MSHA and NPT could reduce tumor growth in a xenograft model of pancreatic cancer.

### PA-MSHA induces pancreatic cancer cell apoptosis *in vivo*

Next, we extracted the total protein from tumor tissue samples to examine the effects of PA-MSHA on apoptosis in the xenograft mice. Interestingly, we observed a reduction in caspase-3 levels in PA-MSHA- compared to PA-treated mice (p < 0.05). In contrast, cleaved caspase-3 levels were higher in PA-MSHA- compared PA-treated mice (p < 0.01; Figure 7). Combined treatment with PA-MSHA and NPT had a synergistic effect on apoptosis (Supplementary Figure 1B). These data indicated that PA-MSHA induced caspase-dependent apoptosis in pancreatic cancer cells *in vivo.*

## DISCUSSION

Pancreatic cancer incidence and mortality have increased in recent years [15]. Surgery is currently the most effective treatment option. However, pancreatic cancer is typically diagnosed at an advanced stage when surgical treatment may no longer be effective. Adjuvant chemotherapy is the standard of care for patients after pancreatic cancer surgery and for patients with advanced-stage disease [16]. Gemcitabine as single-agent chemotherapy has traditionally been the standard treatment regimen, but it has a modest survival benefit [17]. More recently, FOLFIRINOX and Nab-paclitaxel plus gemcitabine have become the standard of care [18–19]. However, poor clinical outcomes are still observed as a result of drug resistance and the side effects of chemotherapy. Therefore, new chemotherapeutic agents are required to improve patient outcomes [20].

In this study, we demonstrated that PA-MSHA inhibited the proliferation of two pancreatic cancer cell lines (PANC-1and SW1990) in a time- and dose-dependent manner. Treatment of pancreatic cancer cells with PA-MSHA resulted in cytoplasmic condensation, cell body shrinkage, nuclear fragmentation, disorganized cellular structures, and the detachment and fusion of the endoplasmic reticulum with the plasma membrane to form multiple vacuoles. These morphological changes indicated PA-MSHA induced apoptosis in pancreatic cancer cells.

Apoptosis was further analyzed in pancreatic cancer cells by flow cytometry. These results confirmed that PA-MSHA induced pancreatic cancer cell apoptosis in a dose-dependent manner. Additionally, we determined that PA-MSHA treatment blocked cell cycle progression at S phase.

EGFR is widely expressed in mammalian epithelial cells. Previous studies have demonstrated higher EGFR [21] and EGF expression in pancreatic cancer tissue compared to normal pancreatic tissue. The expression of EGF/EGFR was correlated with tumor proliferation, invasion, and metastasis [22]. EGFR inhibitors can inhibit the growth and proliferation of tumor cells [23]. Our results indicated that PA-MSHA treatment did not affect the total levels of EGFR signaling pathway components compared to controls. However, the levels of pEGFR, pAKT, and pERK decreased in response to treatment with increasing concentrations of PA-MSHA, suggesting that PA-MSHA inhibited phosphorylation of these key downstream signaling proteins. Thus, PA-MSHA inhibits EGFR signaling and cell proliferation in pancreatic cancer cells.

Caspase-3 functions in the last step of caspase activation and plays an important role in apoptosis [24]. PA has been shown to induce apoptosis through the caspase pathway [25]. However, heat-inactivated PA does not have the capacity to induce apoptosis [26–27]. Consistent with previous reports, we found that inactivated PA did not induce apoptosis in pancreatic cancer cells. However, inactivated PA-MSHA was capable of inducing apoptosis *in vitro*. Therefore, we hypothesized that PA-MSHA could induce caspase-dependent apoptosis in tumor cells *in vivo*. Our data indicated that caspase-3 expression was reduced in pancreatic tumor tissue compared to normal pancreatic tissue, and that the level of cleaved caspase-3 increased in response to PA-MSHA treatment. The combination of PA-MSHA and NPT had a synergistic inhibitory effect on tumor growth. Overall, our data provide insight into the molecular mechanisms underlying PA-MSHA-induced apoptosis in pancreatic cancer cells. Because PA-MSHA inhibits pancreatic cancer cell proliferation and induces apoptosis, it may be a novel treatment for pancreatic cancer that can improve patient prognosis.

## MATERIALS AND METHODS

### Cell culture and reagents

Pancreatic cancer (PANC-1 and SW1990) and normal human pancreatic duct epithelial (HPDE6-C7) cell lines were obtained from the American Type Culture Collection. Cells were maintained in DMEM medium supplemented with 10% FBS (Gibco, USA) at 37°C in a humidified incubator containing 5% CO_2_ (Shanghai Medical Instruments, China). PA-MSHA and wild type PA were obtained from Beijing Wanter Bio-Pharmaceutical (China). The following antibodies were used in this study: anti-pEGFR, -EGFR, -pERK, -ERK, -pAKT, -AKT, -caspase-3, -cleaved caspase-3, and -GAPDH (Cell Signaling Technology, USA).

### CCK-8 assays of cell viability

The cytotoxic effects of PA-MSHA were examined using CCK-8 assays (Cell Counting Kit-8, Dojindo Molecular Technologies Inc., USA). Cells were treated with 0, 0.125, 0.25, 0.5, 1.0, 2.0, 4.0, or 8.0 × 10^9^/mL PA-MSHA and then maintained at 37°C for 24, 48, or 72 hours. The IC_50_ values were calculated using CalcuSyn (Biosoft, USA).

### Transmission electron microscopy

Cells were treated with either 0.5 × 10^9^/mL PA or PA-MSHA for 24 and 48 hours. They were then washed in twice in phosphate-buffered saline (PBS). Cells were fixed in glutaraldehyde for 2 hours, washed three times with PBS, and then fixed in osmium tetroxide for 2 hours. The samples were washed three times in PBS, dehydrated in a stepwise gradient of ethanol, and then embedded in resin. Samples were sectioned, stained, and imaged using a Hitachi Transmission Electron Microscope (Hitachi, Japan).

### Flow cytometric apoptosis assays

Cells were treated with 0.5 × 10^9^/mL PA or 0.25, 0.5, or 1.0 × 10^9^/mL PA-MSHA for 24 hours, and then resuspended in serum-free media at a concentration of 1 × 10^6^/mL. The cells were treated with 5 μL FITC-annexin V and 5 μL propidium iodide (PI; 50 μg/mL), mixed gently, and incubated at room temperature for 15 minutes in the dark. We then added 400 μL 1× Binding Buffer to each sample. Apoptosis was analyzed by flow cytometry using a FACSCalibur system (BD Biosciences, USA). For cell cycle analysis, cells were resuspended as described previously and then fixed in 5 mL of 70% ethanol at 4°C overnight. Samples were then washed twice with PBS and PI added to a final concentration of 50 μg/mL. After incubating the samples at 4°C for 30 minutes, cell cycle profiles were analyzed by flow cytometry.

### Western blotting

Cells were treated with 0.5 × 10^9^/mL PA or 0.25, 0.5, or 1.0 × 10^9^/mL PA-MSHA, and total protein extracted and quantified using BCA assays. Proteins were separated by SDS-PAGE, transferred to PVDF membranes, and incubated with the primary and secondary antibodies listed above. The amount of protein was estimated using the Quantity One software (Bio-Rad, USA) and the relative expression of the target proteins calculated as the amount of the target protein divided by that of GAPDH.

### *In vivo* xenograft studies

Four-week-old female Balb/c-nu/nu nude mice were obtained from the Shanghai Institute of Materia Medica at the Chinese Academy of Sciences. Mice were randomly assigned to either the control or experimental group, and 5 × 10^6^ PANC-1 cells subcutaneously injected into the right hind limbs. Subcutaneous injections of 0.1 mL PA (2 × 10^10^/ml) and 0.1 mL PA-MSHA (2 × 10^10^/mL) were administered daily to the control and experimental groups, respectively. The mice were injected intraperitoneally with Nab-paclitaxel (10 mg/kg, twice a week) for 7 weeks. Tumor size was measured twice a week using a vernier caliper. The following formula was used to calculate tumor size (V): V = π/6 × (W^2^ × L), and the values used to generate tumor growth curves. After the experiment, tumor tissues were collected and proteins analyzed by western blotting.

### Statistical analysis

Statistical analyses were performed using SPSS13.0 (SPSS Inc. Chicago, IL, USA). Data are presented as the mean ± standard deviation ($\bar{\text{x}}\;\pm \text{ SD}$). Differences between the control and experimental groups were analyzed using Student's t tests. Tumor growth curves were analyzed using Mann-Whitney U tests. A *p* value < 0.05 was considered statistically significant.

## SUPPLEMENTARY MATERIALS FIGURE


