# Supplementary material for: Pseudomonas aeruginosa-mannose-sensitive hemagglutinin inhibits pancreatic cancer cell proliferation and induces apoptosis via the EGFR pathway and caspase signaling
Source: Oncotarget. 2016 Oct 24;7(47):77916–25. doi: 10.18632/oncotarget.12844 (PMC5363631; doi:10.18632/oncotarget.12844)
Supplement: Supplementary file 1 [file oncotarget-07-77916-s001.pdf]

Pseudomonas aeruginosa-mannose-sensitive hemagglutinin inhibits pancreatic cancer cell proliferation and induces apoptosis via the EGFR pathway and caspase signaling

SUPPLEMENTARY FIGURE

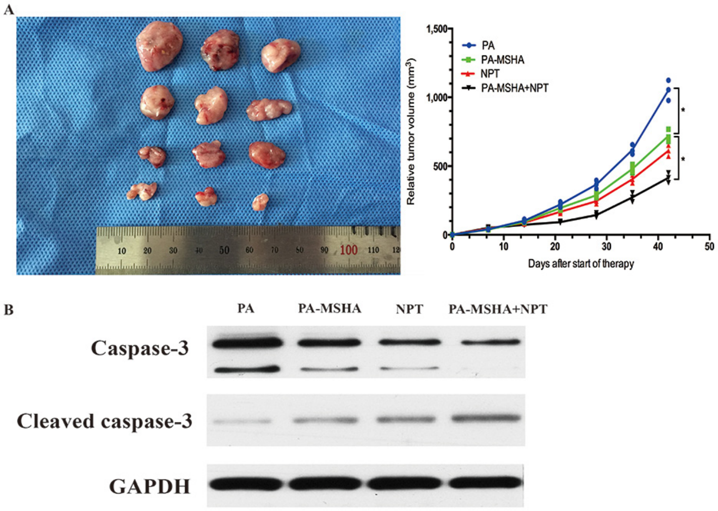

Supplementary Figure 1: Synergistic Inhibitive effect on tumor growth in vivo by PA-MSHA and NPT. A. Tumor volume measured at the indicated time-points. Treatment was initiated 10 days after implantation of pancreatic cancer cells into the mammary fat pads of mice. The mice were injected with PA, PA-MSHA, NPT and PA-MSHA plus NPT for 7 weeks and the tumor volumes measured. *p < 0.01. **B.** Caspase-3 and cleaved caspase-3 expression in xenograft mice treated with PA, PA-MSHA, NPT and PA-MSHA plus NPT.
